# HIF1α-BNIP3-mediated mitophagy protects against renal fibrosis by decreasing ROS and inhibiting activation of the NLRP3 inflammasome

**DOI:** 10.1038/s41419-023-05587-5

**Published:** 2023-03-17

**Authors:** Jialin Li, Qisheng Lin, Xinghua Shao, Shu Li, Xuying Zhu, Jingkui Wu, Shan Mou, Leyi Gu, Qin Wang, Minfang Zhang, Kaiqi Zhang, Jiayue Lu, Zhaohui Ni

**Affiliations:** 1 Department of Nephrology, Molecular Cell Lab for Kidney Disease, Shanghai Peritoneal Dialysis Research Center, Ren Ji Hospital, Uremia Diagnosis and Treatment Center, Shanghai Jiao Tong University School of Medicine, Shanghai, 200127 China; 2 Shuguang Hospital Affilliated to Shanghai University of Traditional Chinese Medicine, Shanghai, 201200 China

**Keywords:** Kidney, Experimental models of disease

## Abstract

Chronic kidney disease affects approximately 14.3% of people worldwide. Tubulointerstitial fibrosis is the final stage of almost all progressive CKD. To date, the pathogenesis of renal fibrosis remains unclear, and there is a lack of effective treatments, leading to renal replacement therapy. Mitophagy is a type of selective autophagy that has been recognized as an important way to remove dysfunctional mitochondria and abrogate the excessive accumulation of mitochondrial-derived reactive oxygen species (ROS) to balance the function of cells. However, the role of mitophagy and its regulation in renal fibrosis need further examination. In this study, we showed that mitophagy was induced in renal tubular epithelial cells in renal fibrosis. After silencing BNIP3, mitophagy was abolished in vivo and in vitro, indicating the important effect of the BNIP3-dependent pathway on mitophagy. Furthermore, in unilateral ureteral obstruction (UUO) models and hypoxic conditions, the production of mitochondrial ROS, mitochondrial damage, activation of the NLRP3 inflammasome, and the levels of αSMA and TGFβ1 increased significantly following BNIP3 gene deletion or silencing. Following silencing BNIP3 and pretreatment with mitoTEMPO or MCC950, the protein levels of αSMA and TGFβ1 decreased significantly in HK-2 cells under hypoxic conditions. These findings demonstrated that HIF1α-BNIP3-mediated mitophagy played a protective role against hypoxia-induced renal epithelial cell injury and renal fibrosis by reducing mitochondrial ROS and inhibiting activation of the NLRP3 inflammasome.

## Introduction

Currently, chronic kidney disease (CKD) is a common disease. CKD affects approximately 14.3% of the population worldwide [1–3]. CKD has not only brought a huge economic burden to patients and nations but has also lowered quality of life. Renal tubulointerstitial fibrosis is the end-stage of almost all CKD [4, 5]. Moreover, the molecular mechanisms of renal tubulointerstitial fibrosis are not completely understood, and it is important to understand the mechanisms to find new therapeutic regimens.

Increasing evidence as shown that renal tubular epithelial cells (RTECs), which are the main cell type of the renal tubules, are closely associated with the state of the kidney. RTECs also play a key role during CKD, driving the progression of interstitial inflammation and fibrosis. Under severe or recurrent injury, RTECs produce various proinflammatory cytokines, including NOD-like receptor and pyrin domain containing-3 (NLRP3). NLRP3 is part of the NOD-like receptor family, which includes pattern recognition receptors [6]. Many studies have shown that elevated levels of NLRP3 and caspase-1 are associated with renal fibrosis in CKD patients [7, 8], indicating that the NLRP3 inflammasome may participate in renal fibrosis. Our previous research showed that MCC950, a specific inhibitor of the NLRP3 inflammasome, can attenuate cisplatin-induced renal fibrosis [9]. Other studies showed an increase in activated NLRP3 inflammasomes in UUO mouse models and 5/6-nephrectomized mice [10, 11]. It has also been reported that the use of anti-IL-1β in the lungs and serum can lower the inflammatory response and attenuate renal fibrosis in mice [12]. In brief, the NLRP3 inflammasome mediates inflammatory responses and participates in the early stage of renal fibrosis. Furthermore, we reported that the NLRP3 inflammasome regulated mitochondrial function and cell apoptosis in CI-AKI models [13].

Recent studies have shown that mitochondria play a crucial role in the activation of the NLRP3 inflammasome. Most ROS are produced by mitochondria. ROS also participate in signaling pathways. Some researchers have indicated that mitochondrial-derived ROS (mtROS) can cause NLRP3 inflammasome activation [14, 15]. In summary, the NLRP3 inflammasome is involved in mitochondrial homeostasis and renal fibrosis.

Mitophagy is a selective pathway to clear damaged mitochondria and has been recognized as an important mechanism for the removal of dysfunctional mitochondria, managing the excessive accumulation of mtROS, and balancing normal cell functions [16, 17]. We have shown that mitophagy protects the kidney in UUO models using PINK1- or PARK2-knockout mice. In UUO models, the level of HIF1α was upregulated [18]. There are two mechanisms of mitophagy. One is the PINK1-PARK2-dependent pathway, and the other is the BNIP3-dependent pathway. Under hypoxic conditions, mitophagy is mediated by BCL-2 and adenovirus E1B 19-kDa-interacting protein3 (BNIP3) or BNIP3-like (BNIP3L), which can directly bind to LC3B to initiate mitophagy [19, 20]. Accumulating evidence has shown that BNIP3 plays a key role in mitophagy. HIF1α is the upstream molecule of BNIP3. It was reported that HIF1α-BNIP3-mediated mitophagy plays a protective role against ischemia/reperfusion-induced acute kidney injury [21]. However, the role of HIF1α-BNIP3-mediated mitophagy in CKD remains unknown. Furthermore, the function of HIF1α-BNIP3-mediated mitophagy and the downstream molecules in renal fibrosis also need to be explored. This study aimed to explore the role of the HIF1α-BNIP3 pathway in mitophagy and the regulation of NLRP3 in renal fibrosis in UUO models by using BNIP3-knockout mice or NLRP3 inflammasome inhibitors.

## Materials and methods

### Cell culture, treatment, transfection with siRNA, antibodies, and reagents

The human renal proximal tubular cell line (HK-2) was purchased from the American Type Culture Collection (ATCC^®^ CRL-2190). HK-2 cells were cultured in DMEM-f12 medium with 10% fetal bovine serum (FBS). To stimulate HK-2 cells with hypoxia, the cells were exposed to 1% oxygen in a hypoxic chamber (Thermo Fisher). All media were equilibrated in a 1% O_2_ atmosphere overnight before use in the hypoxic study. Lipofectamine TM 3000 transfection reagent (Thermo Fisher Scientific, L3000150) was used to transiently transfect HK-2 cells with siRNA (50 nM) for 6 h. The small interfering RNA (siRNA) sequence was as follows: BNIP3 siRNA 5′-CGUUCCAGCCUCGGUUUCUAUUUAU-3′. MitoTEMPO (100 μM, Sigma-Aldrich) and MCC950 (10 μM, MedChemExpress, HY-12815) were added to the culture medium for 4 h before hypoxic exposure [13]. We obtained antibodies targeting LC3 (rabbit, L7543) from Sigma-Aldrich; HIF1α (NB100-105) from Novus Biologicals; BNIP3 (sc-56167), GAPDH (sc-66163), caspase-1 (sc-56036), and TGFβ1 (sc-130348) from Santa Cruz Biotechnology; voltage-dependent anion channel (VDAC) (ab14734) from Abcam; NLRP3 (#15101) from Cell Signal Technology; Caspase-1 (sc-56036) from Santa Cruz Biotechnology; IL-1β (mouse, AF-401-NA; human, AF-201-NA), from R&D Systems; MnSOD (24127-1-AP) and Cyto C (10993-1-AP) from Proteintech; and α-tubulin (AF0001) from Beyotime. The fluorescent secondary antibodies were as follows: donkey anti-rabbit IgG (Alexa Fluor® 555, ab150074; Alexa Fluor® 488, ab150073) and donkey anti-mouse IgG (Alexa Fluor® 488, ab150105; Alexa Fluor® 555, ab150110). MitoTracker Red (M7512) was purchased from ThermoFisher Scientific.

### Animals and the unilateral ureteral obstruction model

Wild-type male C57BL/6 J mice (7-8 weeks old) were purchased from SPF (Beijing) Biotechnology Co., Ltd. BNIP3-knockout mice with a C57BL/6 J background were constructed at Shanghai Research Center of Southern Model Organisms and housed in pathogen-free conditions. First, the mice were anesthetized with pentobarbital sodium. Then, we generated the UUO model. We made a two-point ligation with silk on the left ureter. The mice were sacrificed 7 days after UUO, and the kidneys were harvested. Sham-operated mice were subjected to the sham operation without ligation of the left ureter. All animal experiments were approved by the Animal Care Committee of Ren Ji Hospital, Shanghai Jiao Tong University School of Medicine, and followed the Animal Protocol Committee of Shanghai Jiao Tong University.

### Histopathology, immunohistochemistry and immunostaining of kidneys and cells

Kidney tissues were fixed with 4% paraformaldehyde and embedded in 10% paraffin. Then, the tissues were sectioned at a thickness of 4 μm for Masson’s trichrome staining and were analyzed by microscopy. We analyzed these images with digital image analysis (Ocular 2.0). The fibrotic areas of Masson’s trichrome-stained kidney sections were assessed ImageJ software according to a previous study [22].

Paraffin-embedded kidney sections (4 μm) were deparaffinized, and ethylene diamine tetra acetic acid (1 mM) was used for antigen retrieval. The slides were incubated with LC3B and VDAC antibodies at 4 °C overnight, followed by secondary antibodies. The images were assessed by fluorescence microscopy (ZEISS, Axio Vert A1) and analyzed by digital image analysis. ImageJ was used to analyze the immunofluorescence intensity.

HK-2 cells were seeded on slides in 24-well culture plates for immunostaining. Living HK-2 cells were incubated with MitoTracker Red (500 nM, M7512, Invitrogen) at 37 °C for 15 min to stain mitochondria. Then, slides were fixed with 4% PFA for 1 h, treated with 0.1% Triton X-100 for 5 min at room temperature, and incubated with LC3 antibodies at 4 °C overnight. Then, slides were incubated with secondary antibodies. Next, the slides were observed by fluorescence microscopy at 550 nm for MitoTracker Red and the respective wavelength for the fluorescent secondary antibodies.

### Transmission electron microscopy

Fresh kidneys (1 mm^3^) were harvested in and then processed as described previously [23]. The 70 nm-thick sections were detected by an H-7650 transmission electron microscope (Hitachi, Tokyo, Japan). After identifying representative proximal tubules at low magnification (×3000), high magnification (×7000) was used to identify mitochondria. Each section was independently quantified by two blinded pathologists.

### Mitochondrial isolation

Tissue mitochondrial extraction kits (Beyotime, C3606) were used to isolate mitochondria from the kidney. After the mitochondria and cytoplasm were isolated by differential centrifugation, we kept the fractions in a storage solution with phenylmethylsulfonyl fluoride for immunoblot analysis.

### Analysis of mitochondrial ROS

ROS levels in living HK-2 cells were detected by the indicator MitoSOX Red (M36008, Invitrogen). Cells were incubated with MitoSOX (5 μM) and Hoechst (5 μl/ml, MedChemExpress, HY-15631) for 1 h at 37 °C, and then positive staining was observed under a fluorescence microscope. Two independent technicians were invited to analyze each specimen in a blinded way.

### Immunoblot analysis

Total protein from renal tissue and HK-2 cells was separated by 12% sodium dodecyl sulfate‒polyacrylamide gel electrophoresis, and then the polyvinylidene difluoride membranes were incubated with primary antibodies at 4 °C overnight. ImageJ was used to quantify the immunoblots.

### Statistical analysis

Statistical analysis was conducted using GraphPad software (version 9.0, GraphPad Software, La Jolla, CA). Qualitative data, including immunoblots and images, were representative of at least 3 experiments and are expressed as the means±standard error of the means (SEM). Student’s *t* test was used to assess the differences between two groups. One-way ANOVA was used to analyze the differences among three or more groups, followed by post hoc analysis. *P* < 0.05 was considered significant.

## Results

### HIF1α-BNIP3-dependent mitophagy, activation of the NLRP3 inflammasome and fibrosis were induced in the kidney following UUO

**Fig. 1:**
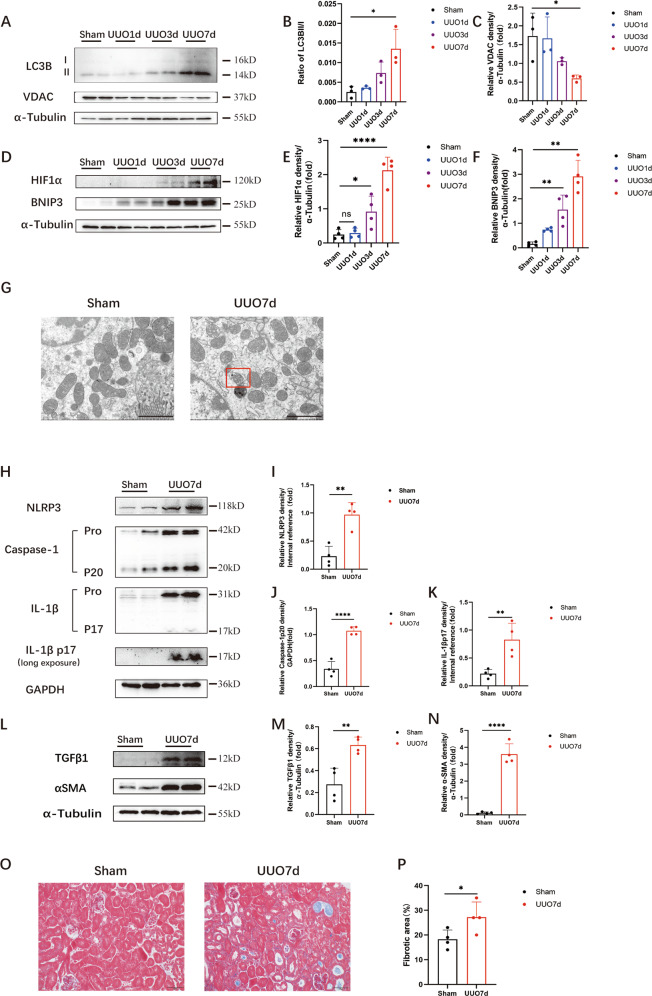
HIF1α-BNIP3-dependent mitophagy, activation of the NLRP3 inflammasome and fibrosis were induced in the kidney following UUO. C57BL/6 J mice were subjected to ligation of left ureter (UUO) or sham operation (Sham). The mice were sacrificed at 1 days, 3 days and 7 days separately. We collected the left kidneys for histological and immunoblot analysis. **A** Immunoblot analysis of LC3B II/I and VDAC in renal tissue and (**B**, **C**) quantification of the protein level of VDAC and ratio of LC3B II/I (*n* = 3 per group). **D** Immunoblot analysis of HIF1α and BNIP3 expression in kidney tissue and (**E,** **F**) quantification of the protein level of HIF1α and BNIP3 (*n* = 4 per group). **G** Representative images of proximal tubular epithelial cells by transmission electron microscopy. Scale bar, 2 μm. In the right panel, the red rectangle indicated an mitophagosome. **H**–**K** Immunoblot analysis and quantification of NLRP3, caspase-1 p20, IL-1βp17 (*n* = 4 per group). **L**–**N** Immunoblot analysis and quantification of αSMA and TGFβ1 (*n* = 4 per group). **O** Representative images of masson’s trichrome staining of kidney sections and (**P**) quantitative analysis of fibrotic area (*n* = 4 per group). Scale bar, 50 μm (×400). Error bars: SEM. *p < 0.05; **p < 0.01; ****p < 0.0001; ns, not significant.

Our previous study showed that mitophagy protected against renal fibrosis in the context of UUO [18]. We found that the autophagy level in mitochondria, as measured by LC3BII levels, was increased markedly in the obstructed kidney. To identify the activation of HIF1α and BNIP3, we first established a UUO model in WT mice. In the WT mouse UUO model, the LC3BII/I ratio rose significantly from 0 d to 7 d. Immunoblot analysis of the mitochondrial outer membranous proteins VDAC and LC3B indicated increased mitophagy in RTECs in the UUO model (Fig. 1A–C). Immunoblot analysis showed that BNIP3 levels were increased significantly after UUO and peaked at 7 d, indicating the activation of BNIP3-dependent mitophagy. Furthermore, immunoblot analysis showed that the protein level of HIF1α, which is the upstream regulator of BNIP3, was increased significantly following UUO (Fig. 1D–F). Compared with that in sham-operated kidneys, transmission electron microscopy (TEM) showed the formation of mitophagosomes in tubular epithelial cells was identified after UUO (Fig. 1G). These results indicated the activation of HIF1α-BNIP3-dependent mitophagy in the context of UUO.

To clarify the effect of the NLRP3 inflammasome on mitochondria in the context of UUO, renal protein levels of NLRP3, cleaved caspase-1 and mature IL-1β were used to assess the activation of the NLRP3 inflammasome. Compared with those in the sham-operation group, these factors were increased significantly in the UUO group (Fig. 1H–K). Moreover, the expression of TGFβ1 and αSMA, which reflect renal fibrosis, was significantly increased (Fig. 1L–N). Furthermore, Masson’s trichrome staining of kidney sections from UUO mice revealed that collagen deposition was increased (Fig. 1O, P). Overall, these data indicated that activation of the NLRP3 inflammasome and fibrosis were induced in the UUO model.

### Hypoxia activated BNIP3-mediated mitophagy, the production of ROS and the NLRP3 inflammasome, as well as fibrosis, in HK-2 cells

**Fig. 2:**
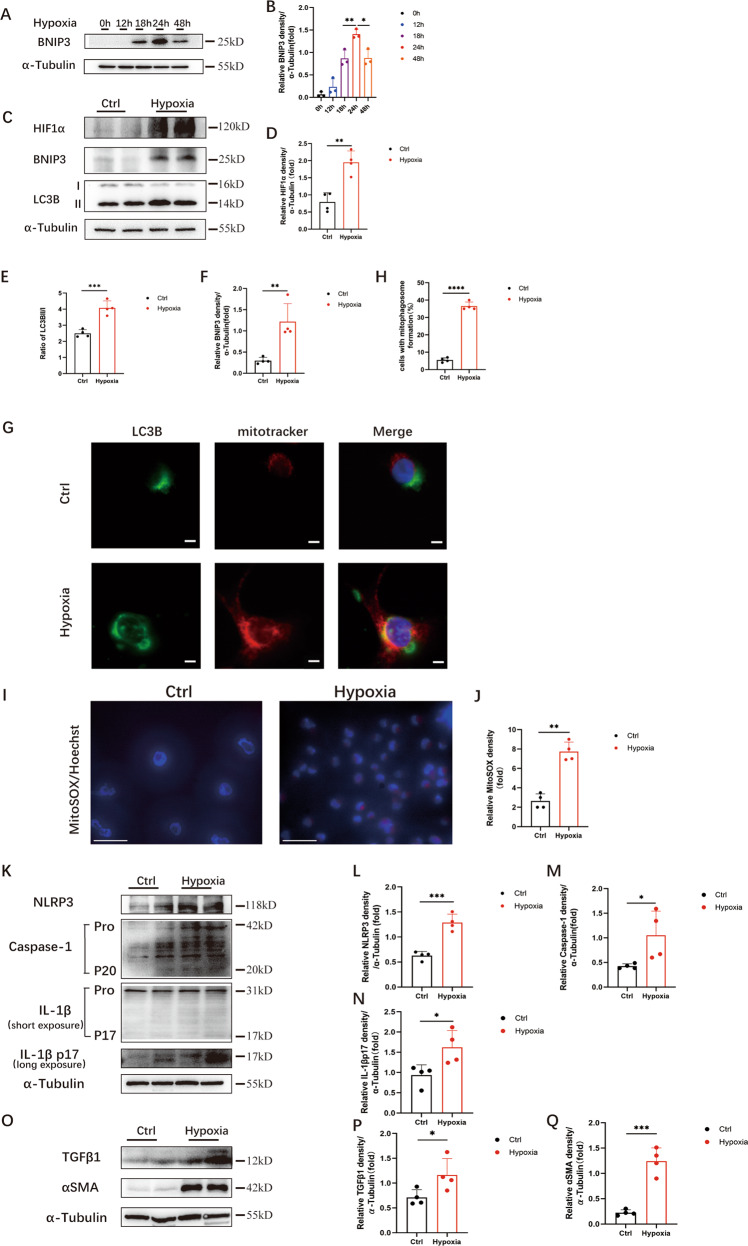
Hypoxia activated BNIP3-mediated mitophagy, the production of ROS and the NLRP3 inflammasome, as well as fibrosis, in HK-2 cells. **A**, **B** Immunoblot analysis and quantification of BNIP3 (*n* = 3 per group). **C** Immunoblot analysis of HIF1α, BNIP3 and LC3BII/I in HK-2 cells in normoxic and hypoxic groups and (**D**–**F**) quantification of the protein levels of HIF1α, BNIP3 and ratio of LC3BII/I (*n* = 4 per group). **G** Representative images of Immunofluorescence of the colocalization of LC3 puncta and mitochondria in HK-2 cells under normoxic and hypoxic conditions and (**H**) quantification data of fluorescence density (*n* = 4 per group). Scale bar, 10 μm. **I** Representative images of MitoSOX/Hoechst staining of HK-2 cells and **J** quantification data of fluorescence density (*n* = 4 per group). Scale bar, 50 μm (**K**) Immunoblot analysis of protein levels and (**L**–**N**) quantification of NLRP3, caspase-1 p20, IL-1β p17 in HK-2 cells (*n* = 4 per group). **O** Immunoblot analysis of protein levels and (**P**, **Q**) quantification of αSMA and TGFβ1 in HK-2 cells (*n* = 4 per group). Error bars: SEM. *p < 0.05; **p < 0.01; ***p < 0.001; ****p < 0.0001.

Accumulating evidence has shown that hypoxia is an early factor in obstruction-induced renal fibrosis. Our previous study confirmed that hypoxia was involved in the activation of mitophagy in the context of UUO [18]. We also demonstrated that the expression of HIF1α increased by 8.5-fold on the 7th day after UUO. To clarify the activation of BNIP3-dependent mitophagy induced by hypoxia, HK-2 cells were cultured in a hypoxic chamber (1% O2) for 6 to 48 h. The cells in the control group were kept in normoxic conditions. Hypoxia induced a significant increase in BNIP3 levels from 18 to 48 h, peaking at 24 h (Fig. 2A, B). Then, we measured the protein levels of HIF1α, BNIP3, LC3BII and LC3BI, which were significantly increased in the hypoxia group of HK-2 cells (Fig. 2C–F). Immunofluorescence analysis showed the colocalization of LC3B puncta and MitoTracker, which indicated the formation of mitophagosomes in HK-2 cells under hypoxic conditions. Compared to that in the control group, the portion of cells with mitophagosome formation increased significantly (Fig. 2G, H).

Most cellular ROS are produced by damaged mitochondria. Therefore, we examined the production of ROS in RTECs in vitro to evaluate the role of BNIP3-dependent mitophagy. Hypoxia-induced HK-2 cells exhibited an increase in the fluorescence intensity of MitoSOX by 3.4-fold compared with HK-2 cells in the control group (Fig. 2I, J). Furthermore, NLRP3 inflammasome activation was verified by immunoblot analysis, and the protein levels of NLRP3, cleaved caspase-1, and mature IL-1β were upregulated by hypoxia (Fig. 2K–N). Similarly, the expression of αSMA and TGFβ1 was increased significantly compared with that in the control group (Fig. 2O–Q). These data suggest that hypoxia can activate BNIP3-dependent mitophagy, the production of ROS, the NLRP3 inflammasome and fibrosis in HK-2 cells.

### BNIP3 deficiency enhanced mitochondrial damage and the release of cytochrome C following UUO

**Fig. 3:**
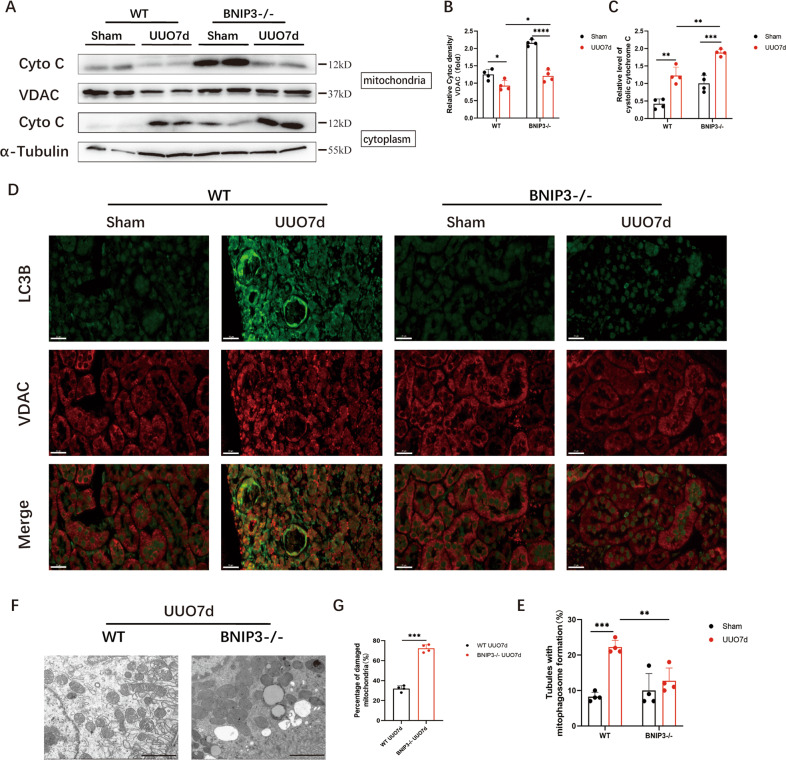
BNIP3 deficiency enhanced mitochondrial damage and the release of cytochrome C following UUO. **A**–**C** Immunoblot analysis and quantification of cytochrome C in cytosolic and mitochondrial fraction (*n* = 4 per group). **D**, **E** Representative images of immunofluorescence double-labelling LC3B and mitochondrial outer membrane protein (VDAC) in sham-operated kidneys or obstructed kidneys in WT and BNIP3-/- mice (*n* = 4 per group). Scale bar, 20 μm. **F** Representative images of proximal tubular epithelial cells by transmission electron microscopy and **G** quantification data of damaged mitochondria in different groups of mice: WT and BNIP3 knockout (*n* = 4 per group). Scale bar, 2 μm (×7000). Error bars: SEM. *p < 0.05; **p < 0.01; ***p < 0.001; ****p < 0.0001.

To clarify the effect of BNIP3-dependent mitophagy on UUO, we used BNIP3-KO mice in this study. First, we examined cytosolic cytochrome C, an important parameter of apoptosis after mitochondrial injury that is released from mitochondria. Immunoblotting showed that BNIP3-KO mice released more cytochrome C into the cytosolic fraction than the WT mice following UUO (Fig. 3A–C). Next, the costaining of VDAC and LC3B suggested the occurrence of mitophagy. However, the costaining of VDAC and LC3B in the BNIP3-KO mouse group was decreased significantly, showing decreased degradation of injured mitochondria via mitophagy because of BNIP3 deficiency (Fig. 3D, E). Third, we examined mitochondrial morphology by TEM in the RTECs of WT and BNIP3-KO mice. The number of mitochondria decreased, and moderate structural damage to mitochondria was observed, including swelling, broken cristae and vacuoles in the mitochondrial matrix following UUO, and these effects were more prominent in BNIP3-KO mice (Fig. 3F, G). After the deletion of BNIP3, the increases in mitochondrial structural damage and cytochrome C levels and the decrease in mitophagy indicated that BNIP3-mediated mitophagy was crucial for protecting RTECs and clearing damaged mitochondria.

### BNIP3 deficiency decreased BNIP3-mediated mitophagy and enhanced mitochondrial ROS production in hypoxia-induced HK-2 cells

**Fig. 4:**
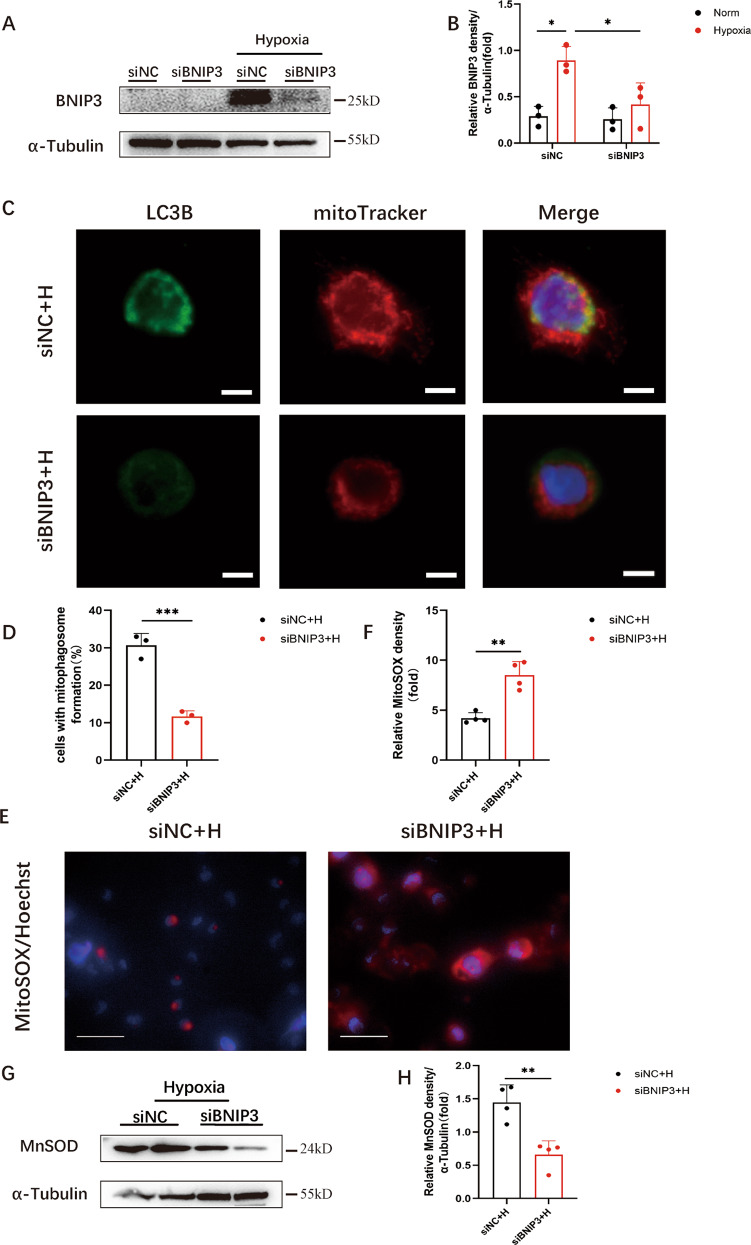
BNIP3 deficiency decreased BNIP3-mediated mitophagy and enhanced mitochondrial ROS production in hypoxia-induced HK-2 cells. **A** After transfected by negative control siRNA or BNIP3 siRNA, HK-2 cells were under the condition of hypoxia for 24 h. Immunoblot analysis and **B** quantification of BNIP3 (*n* = 3 per group). **C**, **D** Representative images and quantification of immunofluorescence double-labelling LC3B and mitochondrial marker (Mitotracker) (*n* = 3 per group). Scale bar: 10 μm. **E** Representative images of MitoSOX/Hoechst staining of HK-2 cells and **F** quantification data of fluorescence density (*n* = 4 per group). Scale bar, 50 μm (×400) **G** Immunoblot analysis of MnSOD in lysates of HK-2 cells, and **H** quantitative data of immunoblotting (*n* = 4 per group). Error bars: SEM. *p < 0.05; **p < 0.01; ***p < 0.001. siNC+H, siNC+Hypoxia. siBNIP3 + H, siBNIP3+Hypoxia.

We used siRNA to silence BNIP3 in HK-2 cells to identify the function of BNIP3-mediated mitophagy in hypoxia-induced HK-2 cells. Immunoblot analysis showed that the protein expression level of BNIP3 decreased significantly in the siBNIP3+hypoxia group (Fig. 4A, B). Furthermore, following hypoxia, the colocalization of LC3B and MitoTracker showed a reduction in the formation of mitophagosomes after silencing BNIP3 (Fig. 4C, D). These data indicated that BNIP3-mediated mitophagy was activated under hypoxic conditions and inhibited by silencing BNIP3.

After silencing the BNIP3 gene in HK-2 cells, HK-2 cells were exposed to hypoxia for 24 h. Consistent with previous immunoblot analysis, the expression of BNIP3 was successfully inhibited by siRNA transfection. Under hypoxic conditions for 24 h, BNIP3 siRNA-treated HK-2 cells exhibited a 2-fold increase in the fluorescence intensity of MitoSOX compared with HK-2 cells in the siNC+hypoxia group (Fig. 4E, F). Furthermore, we measured MnSOD levels. MnSOD is a specific type of SOD that is located in mitochondria. Usually, the activity of MnSOD reveals the capacity of cells to remove mitochondrial superoxide. Immunoblot analysis showed that compared with that in the siNC+hypoxia group, the production of MnSOD protein decreased significantly in the siBNIP3+hypoxia group (Fig. 4G, H).

Overall, these data demonstrated that mitochondrial ROS production was increased in the siBNIP3+hypoxia group due to hypoxia, indicating that HIF1α-BNIP3-mediated mitophagy restricted the production of mitochondrial ROS in HK-2 cells due to hypoxia.

### BNIP3 deficiency enhanced NLRP3 inflammasome activation and aggravated renal fibrosis after UUO

**Fig. 5:**
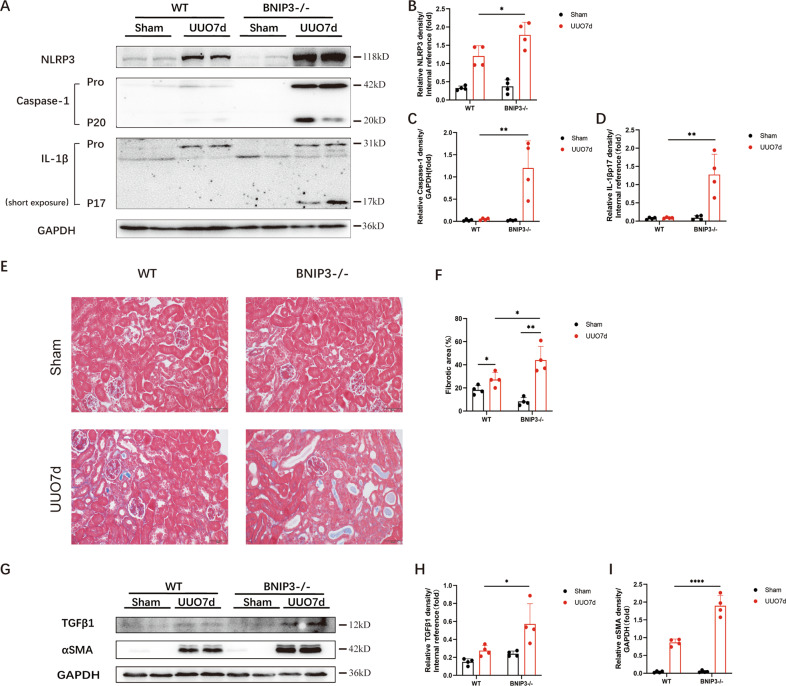
BNIP3 deficiency enhanced NLRP3 inflammasome activation and aggravated renal fibrosis after UUO. WT and BNIP3 knockout mice were subjected to sham-operation or ligation of the left ureter, we collected the left kidney at 7 days after UUO. **A** Immunoblot analysis of protein levels and **B**–**D** quantification of NLRP3, caspase-1 p20, IL-1β p17 in kidney tissues (*n* = 4 per group). **E** Representative images of masson’s trichrome staining of kidney sections and **F** quantitative analysis of fibrotic area (*n* = 4 per group). Scale bar, 50 μm (×400). **G** Immunoblot analysis of protein levels and **H**, **I** quantification of αSMA and TGFβ1 in kidney tissues (*n* = 4 per group). Error bars: SEM. *p < 0.05; **p < 0.01; ****p < 0.0001.

It was reported that mitophagy inhibited activation of the NLRP3 inflammasome to attenuate apoptosis in AKI by limiting the release of ROS [13]. To date, the actual role of BNIP3-mediated mitophagy in renal fibrosis remains unknown. In WT mice, we confirmed that the NLRP3 inflammasome was activated following UUO. Furthermore, immunoblot analysis of the NLRP3 inflammasome demonstrated that BNIP3-KO mice in the UUO group had higher protein levels of NLRP3, cleaved caspase-1 and mature IL-1β than those in the WT group (Fig. 5A–D). In addition, BNIP3-KO mice showed more collagen deposition in the tubular interstitium and more severe tubular injury by Masson’s trichrome staining following UUO (Fig. 5E, F). Moreover, BNIP3-KO mice showed a higher level of TGFβ1 in tubular epithelial cells than WT mice in the UUO group. The expression of αSMA in kidney tissue was also significantly increased in BNIP3-KO mice compared to WT mice (Fig. 5G–I). Therefore, these findings suggest that BNIP3-mediated mitophagy plays a crucial role in attenuating renal fibrosis.

### NLRP3 inflammasome activation and fibrosis were induced after silencing BNIP3 in hypoxia-treated HK-2 cells

**Fig. 6:**
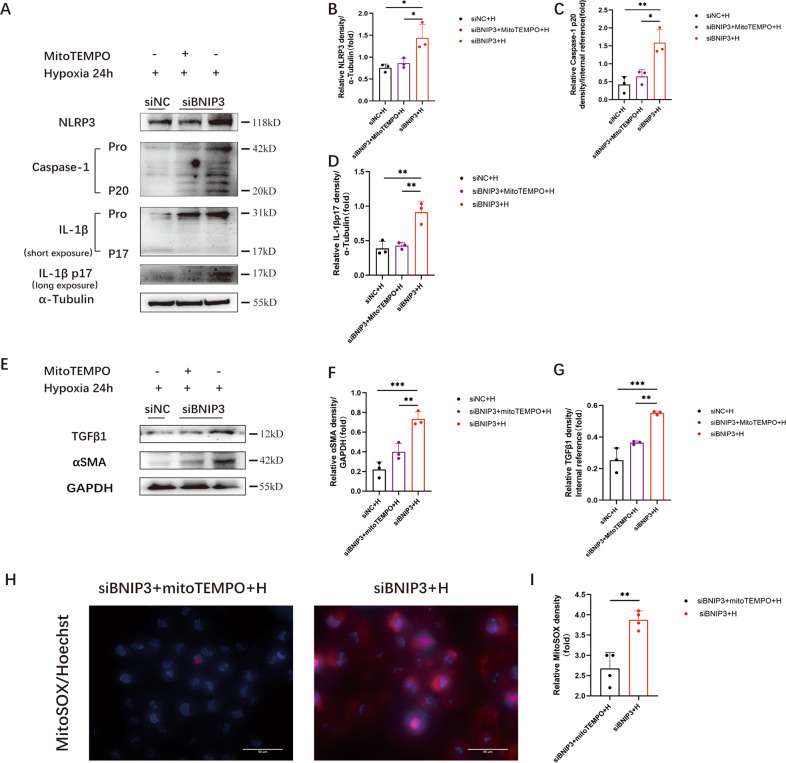
NLRP3 inflammasome activation and fibrosis were induced after silencing BNIP3 in hypoxia-treated HK-2 cells. After transfected by siNC or siBNIP3 for 6 h, HK-2 cells were pretreated with MitoTEMPO (100 μM) for 4 h and then cultured under the hypoxic conditions for 24 h. **A** Immunoblot analysis of protein levels and **B**–**D** quantification of NLRP3, caspase-1 p20, IL-1β p17 in HK-2 cells. (*n* = 3 per group) **E** Immunoblot analysis of protein levels and **F**, **G** quantification of αSMA and TGFβ1 in HK-2 cells (*n* = 3 per group). **H** Representative images of MitoSOX/Hoechst staining of HK-2 cells and **I** quantification data of fluorescence density (*n* = 4 per group). Scale bar, 50 μm (×400). Error bars: SEM. *p < 0.05; **p < 0.01; ***p < 0.001.

As described previously, we found that BNIP3 siRNA-treated HK-2 cells exhibited increased fluorescence intensity of MitoSOX compared with HK-2 cells in the siNC+hypoxia group. Furthermore, MnSOD levels in the siBNIP3+hypoxia group were more significantly reduced than those in the siNC+hypoxia group in HK-2 cells. BNIP3 deficiency markedly reduced the activity of the SOD enzyme in mitochondria under hypoxic conditions following inhibition of the BNIP3-mediated mitophagy pathway. In addition, we used immunoblot analysis to examine NLRP3 inflammasome activation. The results showed that NLRP3, cleaved caspase-1, and mature IL-1β were increased significantly in the hypoxia-treated group after BNIP3 silencing (Fig. 6A–D). MitoTEMPO is a mitochondria-targeted antioxidant. HK-2 cells were transfected with BNIP3 siRNA and pretreated with 100 μM MitoTEMPO for 4 h. Immunoblot analysis showed that MitoTEMPO attenuated the overactivation of the NLRP3 inflammasome (Fig. 6A–D). Immunoblot analysis was used to examine αSMA and TGFβ1. The expression of αSMA and TGFβ1 was increased significantly following BNIP3 silencing in the hypoxia-induced group compared with the siNC +hypoxia group (Fig. 6E–G). In addition, immunoblot analysis showed that the expression of αSMA and TGFβ1 decreased significantly in HK-2 cells that were pretreated with MitoTEMPO (Fig. 6E–G). Moreover, under hypoxic conditions for 24 h, BNIP3 siRNA-treated HK-2 cells exhibited an increase in the fluorescence intensity of MitoSOX by 1.4-fold compared with HK-2 cells in the MitoTEMPO-pretreated group (Fig. 6H, I).

These data indicate that BNIP3-mediated mitophagy deficiency can cause NLRP3 inflammasome activation and aggravate fibrosis. MitoTEMPO, the specific antioxidant for mtROS, can attenuate the increased fibrosis induced by hypoxia in HK-2 cells.

### NLRP3 inflammasome inhibition alleviated hypoxia-induced fibrosis in HK-2 cells after silencing BNIP3

**Fig. 7:**
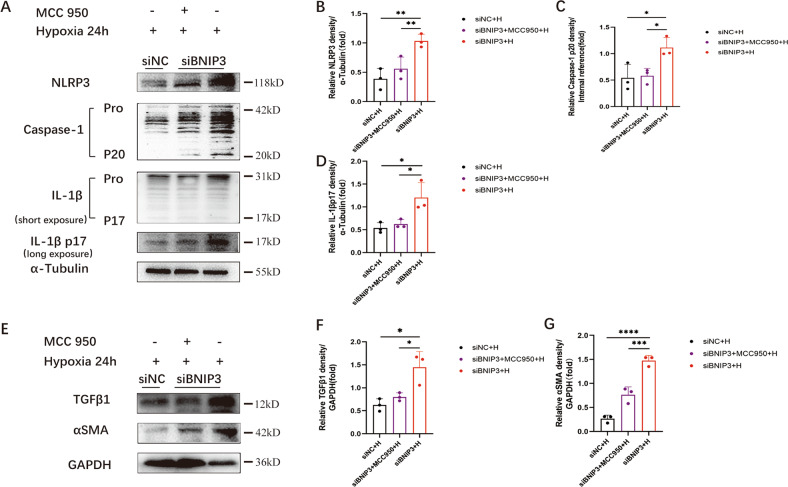
NLRP3 inflammasome inhibition alleviated hypoxia-induced fibrosis in HK-2 cells after silencing BNIP3. After transfected by siNC or siBNIP3 for 6 h, HK-2 cells were pretreated with MCC950 (10 μM) for 4 h and then cultured under the hypoxic conditions for 24 h. **A** Immunoblot analysis of protein levels and **B**–**D** quantification of NLRP3, caspase-1 p20, IL-1β p17 in HK-2 cells (*n* = 3 per group). **E** Immunoblot analysis of protein levels and **F**, **G** quantification of αSMA and TGFβ1 in HK-2 cells (*n* = 3 per group). Error bars: SEM. *p < 0.05; **p < 0.01; ***p < 0.001; ****p < 0.0001.

In this study, we explored the regulatory role of HIF1α-BNIP3-mediated mitophagy in renal fibrosis. Immunoblot analysis showed an increase in αSMA and TGFβ1 in response to hypoxia exposure following BNIP3 silencing. To investigate the relationship between the NLRP3 inflammasome and hypoxia-induced fibrosis, after being transfected with siBNIP3, HK-2 cells were pretreated with MCC950 to inhibit NLRP3 inflammasome activation before being exposed to hypoxia (Fig. 7A–D). Hypoxia-induced fibrosis following BNIP3 silencing was abolished by MCC950, as demonstrated by immunoblot analysis of αSMA and TGFβ1 in the MCC950 group (Fig. 7E–G).

Therefore, HIF1α-BNIP3-mediated mitophagy can protect HK-2 cells from fibrosis by inhibiting the NLRP3 inflammasome by reducing the production of ROS.

## Discussion

In the present study, we confirmed that the tubular HIF1α-BNIP3 signaling pathway is involved in mitophagy and protects against renal fibrosis. We found that HIF1α, which is an upstream molecule of BNIP3, was activated in renal tubular cells in the context of UUO and HK-2 cells under hypoxic conditions. Next, we demonstrated that BNIP3-mediated mitophagy was activated under the same conditions. Then, we showed the function of BNIP3-mediated mitophagy in UUO by examining BNIP3-KO mice and siRNA in HK-2 cells. BNIP3 deficiency increased the production of mitochondrial ROS, the NLRP3 inflammasome, and αSMA and TGFβ1 expression, indicating the protective role of BNIP3-mediated mitophagy in renal fibrosis. Furthermore, we demonstrated that renal fibrosis was attenuated by decreasing mitochondrial ROS and NLRP3 inflammasome production and TGFβ1 activation with MitoTEMPO, a selective mitochondrial-targeted antioxidant, and MCC950, a specific NLRP3 inhibitor.

Accumulating evidence indicates that the levels of NLRP3 are increased significantly in the kidneys of CKD [7, 8]. In UUO mouse models, the levels of phosphorylated NF-κB and the NLRP3 inflammasome were elevated, and renal matrix accumulation was increased [10, 11]. NLRP3 activators can induce the production of ROS, and then ROS can activate the NLRP3 inflammasome. It was reported that NLRP3 deletion could reverse the damage to mitochondria in the context of UUO and mitigate renal fibrosis [24]. It was reported that NLRP3 can play a role in interstitial fibrosis in renal tubular epithelial cells, including regulating NLRP3 and TGF-β signaling in renal tubular epithelial cells [25]. Romero showed that hyperuric acid upregulates the expression of NLRP3/ASC and triggers the inflammation-associated caspase-1 and inflammation-independent smad2/3 pathway. Ultrastructural colocalization of NLRP3 and smad2/3 suggests an interaction between these factors [26]. In our study, we found that mitoTEMPO, a mitochondrial antioxidant agent, and MCC950, a specific NLRP3 inhibitor, could downregulate the expression of the NLRP3 inflammasome and alleviate renal fibrosis in the siBNIP3+hypoxia group in HK-2 cells.

Accumulating evidence has shown that mitophagy plays a crucial role in eliminating damaged and dysfunctional mitochondria and maintaining cellular homeostasis [27]. To date, most researchers have focused on mitophagy in AKI. According to our previous research on CI-AKI, mitochondrial ROS and oxidized DNA trigger the NLRP3 inflammasome [13]. Furthermore, our previous study showed that inhibiting BNIP3-mediated mitophagy increased mortality, renal injury and apoptosis in CI-AKI mice [23]. Tang et al. suggested that BNIP3-mediated mitophagy played a protective role in renal ischemia‒reperfusion injury. The loss of BNIP3 resulted in the accumulation of damaged mitochondrial fragments, mitochondrial ROS and inflammatory responses after renal IR [28]. Liang et al. found that *Panax notoginseng* saponins could mitigate cisplatin-induced nephrotoxicity by activating the HIF1α-BNIP3 signaling pathway [29]. To date, there has been no research on the effect of BNIP3-mediated mitophagy in CKD. In our study, there was more severe renal fibrosis, higher levels of mitochondrial ROS, and increased production of the NLRP3 inflammasome in BNIP3-KO mice following UUO. Our present study supports the renoprotective role of BNIP3-mediated mitophagy in the context of UUO.

**Fig. 8:**
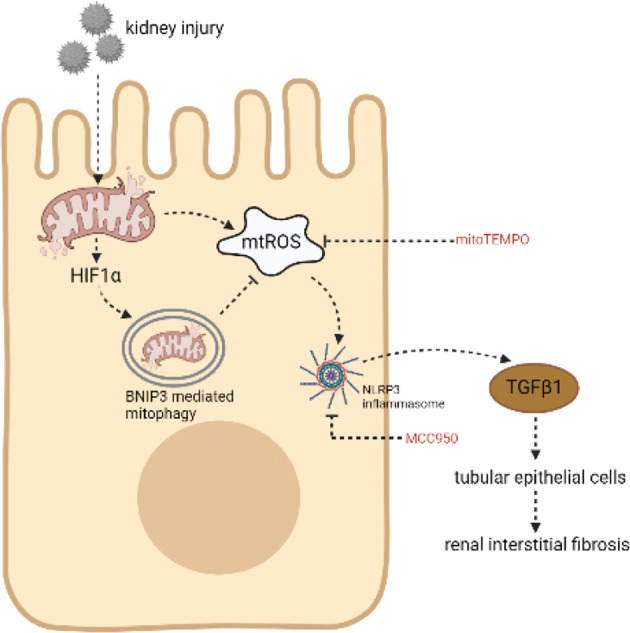
Proposed mechanism for HIF1α-BNIP3-mediated mitophagy in alleviating renal fibrosis in UUO. Following UUO, a huge amount of ROS was released from damaged mitochondria, leading to increased expression of TGFβ1. BNIP3 deficiency enhanced the NLRP3 inflammasome activation and aggravated renal fibrosis in vivo and in vitro. Both inhibition of NLRP3 inflammasome and clearing ROS could protect the kidney against fibrosis, indicating the protective role of HIF1α-BNIP3-mediated mitophagy in UUO by eliminating ROS and then inhibiting activation of the NLRP3 inflammasome.

Mitochondria are important for maintaining the homeostasis of cells. Damaged mitochondria can delay kidney repair [30]. Timely removal of damaged mitochondria benefits kidney repair. Accumulating evidence has indicated the role of tubular epithelial cells in renal fibrosis [31, 32]. In the progression of AKI to CKD, apoptosis in renal tubular cells after renal injury is the first step and is relevant to tubulointerstitial fibrosis [33, 34]. Su et al. suggested that NLRP3 regulated mitophagy in an inflammasome-independent manner in renal tubular cells under hypoxic conditions. Activated NLRP3 can negatively regulate mitophagy [35]. Our previous research revealed the role of the mitophagy-mitochondrial ROS NLRP3 inflammasome pathway in CI-AKI [13]. In the present study, we demonstrated that HIF1α-BNIP3-mediated mitophagy played an important role in regulating the NLRP3 inflammasome in the context of UUO. The deletion of BNIP3 decreased the activity of MnSOD, increased mitochondrial ROS and activated the NLRP3 inflammasome and renal fibrosis following UUO (Fig. 8). Furthermore, renal fibrosis was attenuated by MitoTEMPO and MCC950, indicating that HIF1α-BNIP3-mediated mitophagy protected against renal fibrosis by regulating the NLRP3 inflammasome.

In this study, we showed that the tubular HIF1α-BNIP3 signaling pathway is involved in mitophagy and plays a protective role in renal fibrosis. However, our study was conducted in an animal model and HK-2 cells. Studies in human beings are currently ongoing.

In summary, we demonstrated that the HIF1α-BNIP3 mitophagy pathway was induced in the context of hypoxia and after UUO. HIF1α-BNIP3-mediated mitophagy protected RTECs against hypoxia and renal tissue following UUO by decreasing mitochondrial ROS and inhibiting activation of the NLRP3 inflammasome to attenuate renal fibrosis. Severe renal fibrosis following UUO caused by BNIP3 deficiency could also be partially mitigated by mitochondria-targeted antioxidants. Thus, therapeutic strategies targeting HIF1α- and BNIP3-mediated mitophagy may alleviate renal fibrosis and delay the progression of CKD.

## Supplementary information

Original Data File

## Data Availability

The authors declare that all data in the article is available.

## References

[1] Ene-Iordache B, Perico N, Bikbov B, Carminati S, Remuzzi A, Perna A (2016). Chronic kidney disease and cardiovascular risk in six regions of the world (ISN-KDDC): a cross-sectional study. Lancet. Glob Health.

[2] Romagnani P, Remuzzi G, Glassock R, Levin A, Jager KJ, Tonelli M (2017). Chronic kidney disease. Nat Rev Dis Prim.

[3] Glassock RJ, Warnock DG, Delanaye P (2017). The global burden of chronic kidney disease: estimates, variability and pitfalls. Nat Rev Nephrol.

[4] Huynh P, Chai Z (2019). Transforming growth factor beta (TGFbeta) and related molecules in chronic kidney disease (CKD). Clin Sci.

[5] Webster AC, Nagler EV, Morton RL, Masson P (2017). Chronic Kidney Disease. Lancet..

[6] Lorenz G, Darisipudi MN, Anders HJ (2014). Canonical and non-canonical effects of the NLRP3 inflammasome in kidney inflammation and fibrosis. Nephrol Dial Transpl.

[7] Vilaysane A, Chun J, Seamone ME, Wang W, Chin R, Hirota S (2010). The NLRP3 inflammasome promotes renal inflammation and contributes to CKD. J Am Soc Nephrol.

[8] Ke B, Shen W, Fang X, Wu Q (2018). The NLPR3 inflammasome and obesity-related kidney disease. J Cell Mol Med.

[9] Li S, Lin Q, Shao X, Mou S, Gu L, Wang L (2019). NLRP3 inflammasome inhibition attenuates cisplatin-induced renal fibrosis by decreasing oxidative stress and inflammation. Exp Cell Res.

[10] Seo JB, Choi YK, Woo HI, Jung YA, Lee S, Lee S (2019). Gemigliptin Attenuates Renal Fibrosis Through Down-Regulation of the NLRP3 Inflammasome. Diabetes Metab J..

[11] Wen Y, Pan MM, Lv LL, Tang TT, Zhou LT, Wang B (2019). Artemisinin attenuates tubulointerstitial inflammation and fibrosis via the NF-kappaB/NLRP3 pathway in rats with 5/6 subtotal nephrectomy. J Cell Biochem.

[12] Guo J, Shi T, Cui X, Rong Y, Zhou T, Zhang Z (2016). Effects of silica exposure on the cardiac and renal inflammatory and fibrotic response and the antagonistic role of interleukin-1 beta in C57BL/6 mice. Arch Toxicol.

[13] Lin Q, Li S, Jiang N, Shao X, Zhang M, Jin H (2019). PINK1-parkin pathway of mitophagy protects against contrast-induced acute kidney injury via decreasing mitochondrial ROS and NLRP3 inflammasome activation. Redox Biol.

[14] Chiu PJ, Vemulapalli S, Sabin C, Rivelli M, Bernardino V, Sybertz EJ (1992). Sympathoadrenal stimulation, not endothelin, plays a role in acute pressor response to cyclosporine in anesthetized rats. J Pharm Exp Ther.

[15] Li N, Ragheb K, Lawler G, Sturgis J, Rajwa B, Melendez JA (2003). Mitochondrial complex I inhibitor rotenone induces apoptosis through enhancing mitochondrial reactive oxygen species production. J Biol Chem.

[16] Zeb A, Choubey V, Gupta R, Kuum M, Safiulina D, Vaarmann A (2021). A novel role of KEAP1/PGAM5 complex: ROS sensor for inducing mitophagy. Redox Biol.

[17] Ni HM, Williams JA, Ding WX (2015). Mitochondrial dynamics and mitochondrial quality control. Redox Biol.

[18] Li S, Lin Q, Shao X, Zhu X, Wu J, Wu B (2020). Drp1-regulated PARK2-dependent mitophagy protects against renal fibrosis in unilateral ureteral obstruction. Free Radic Biol Med.

[19] Yuan Y, Zheng Y, Zhang X, Chen Y, Wu X, Wu J (2017). BNIP3L/NIX-mediated mitophagy protects against ischemic brain injury independent of PARK2. Autophagy..

[20] Quinsay MN, Thomas RL, Lee Y, Gustafsson AB (2010). Bnip3-mediated mitochondrial autophagy is independent of the mitochondrial permeability transition pore. Autophagy..

[21] Fu ZJ, Wang ZY, Xu L, Chen XH, Li XX, Liao WT (2020). HIF-1alpha-BNIP3-mediated mitophagy in tubular cells protects against renal ischemia/reperfusion injury. Redox Biol.

[22] Rutkowski JM, Wang ZV, Park AS, Zhang J, Zhang D, Hu MC (2013). Adiponectin promotes functional recovery after podocyte ablation. J Am Soc Nephrol.

[23] Lin Q, Li S, Jiang N, Jin H, Shao X, Zhu X (2021). Inhibiting NLRP3 inflammasome attenuates apoptosis in contrast-induced acute kidney injury through the upregulation of HIF1A and BNIP3-mediated mitophagy. Autophagy..

[24] Guo H, Bi X, Zhou P, Zhu S, Ding W (2017). NLRP3 Deficiency Attenuates Renal Fibrosis and Ameliorates Mitochondrial Dysfunction in a Mouse Unilateral Ureteral Obstruction Model of Chronic Kidney Disease. Mediators Inflamm.

[25] Wang W, Wang X, Chun J, Vilaysane A, Clark S, French G (2013). Inflammasome-independent NLRP3 augments TGF-beta signaling in kidney epithelium. J Immunol.

[26] Romero CA, Remor A, Latini A, De Paul AL, Torres AI, Mukdsi JH (2017). Uric acid activates NRLP3 inflammasome in an in-vivo model of epithelial to mesenchymal transition in the kidney. J Mol Histol.

[27] Tang C, He L, Liu J, Dong Z (2015). Mitophagy: Basic Mechanism and Potential Role in Kidney Diseases. Kidney Dis..

[28] Tang C, Han H, Liu Z, Liu Y, Yin L, Cai J (2019). Activation of BNIP3-mediated mitophagy protects against renal ischemia-reperfusion injury. Cell Death Dis.

[29] Liang X, Yang Y, Huang Z, Zhou J, Li Y, Zhong X (2017). Panax notoginseng saponins mitigate cisplatin induced nephrotoxicity by inducing mitophagy via HIF-1alpha. Oncotarget..

[30] Qin J, Peng ZZ, Li Q, Wen R, Tao LJ (2018). Renal Fibrosis and Mitochondrial Damage. Chin Med J.

[31] Liu BC, Tang TT, Lv LL, Lan HY (2018). Renal tubule injury: a driving force toward chronic kidney disease. Kidney Int.

[32] Yang L, Besschetnova TY, Brooks CR, Shah JV, Bonventre JV (2010). Epithelial cell cycle arrest in G2/M mediates kidney fibrosis after injury. Nat Med..

[33] Zhao Y, Sun X, Nie X, Sun L, Tang TS, Chen D (2012). COX5B regulates MAVS-mediated antiviral signaling through interaction with ATG5 and repressing ROS production. PLoS Pathog.

[34] Sun X, Sun L, Zhao Y, Li Y, Lin W, Chen D (2016). MAVS maintains mitochondrial homeostasis via autophagy. Cell Disco.

[35] Kim SM, Kim YG, Kim DJ, Park SH, Jeong KH, Lee YH (2018). Inflammasome-Independent Role of NLRP3 Mediates Mitochondrial Regulation in Renal Injury. Front Immunol.

